# Seasonal variation in the xylem sap composition of six Australian trees and shrubs

**DOI:** 10.1093/aobpla/plad064

**Published:** 2023-09-12

**Authors:** Adriano Losso, Alice Gauthey, Brendan Choat, Stefan Mayr

**Affiliations:** Hawkesbury Institute for the Environment, Western Sydney University, Locked Bag 1797 Penrith, 2751 New South Wales, Australia; Department of Botany, University of Innsbruck, Sternwartestraße 15, 6020 Innsbruck, Austria; Hawkesbury Institute for the Environment, Western Sydney University, Locked Bag 1797 Penrith, 2751 New South Wales, Australia; Plant Ecology Research Laboratory PERL, Ecole Polytechnique Fédérale de Lausanne EPFL, 1015 Lausanne, Switzerland; Swiss Federal Institute for Forest, Snow and Landscape Research WSL, Zürcherstrasse 111, 8903 Birmensdorf, Switzerland; Hawkesbury Institute for the Environment, Western Sydney University, Locked Bag 1797 Penrith, 2751 New South Wales, Australia; Department of Botany, University of Innsbruck, Sternwartestraße 15, 6020 Innsbruck, Austria

**Keywords:** Australian shrubs, Australian trees, calcium, drought, ions, pH, seasonal course, xylem sap

## Abstract

In recent years, xylem sap composition has been shown to affect xylem hydraulics. However, information on how much xylem sap composition can vary across seasons and specifically under drought stress is still limited. We measured xylem sap chemical composition ([Ca^2+^], [K^+^], [Na^+^], electrical conductivity EC and pH) and surface tension (*γ*) of six Australian angiosperm trees and shrubs over 1 year, which comprised of exceptional dry and wet periods. Percentage losses of hydraulic conductivity and predawn leaf water potential were also monitored. In all species, measured parameters changed considerably over the annual time course. Ions and pH tended to decrease during winter months whereas *γ* showed a slight increase. No clear correlation was found between sap and hydraulic parameters, except for pH that was higher when plants suffered higher drought stress levels. Results indicate xylem sap composition to be complex and dynamic, where most variation in its composition seems to be dictated by season, even under severe dry conditions. However, pH might play a role as signals of drought stress.

## Teaser

The composition of xylem sap has been shown to affect the water status of plants, but little is known on how much it can vary across seasons and specifically under drought stress. In this study, we measured xylem sap composition of six Australian angiosperm trees and shrubs over one year, which comprised exceptional dry and wet periods. The water status of these plants was also monitored. In all species, xylem sap composition considerably changed across seasons, and no clear correlation between xylem sap and plants water status was found. Results indicate xylem sap composition to be complex and dynamic, where most variation in its composition seems to be dictated by seasons, even under severe dry conditions. However, pH was the only parameter which was higher when plants suffered higher drought stress levels and might thus play a role as signals of drought stress.

## Introduction

In vascular plants, water is transported from roots to leaves through the xylem, a network consisting of many interconnected conduits. According to the cohesion-tension theory (Boehm 1893; Dixon and Joly 1895; Steudle 2001), transpiration at the leaf surface generates a water potential (*Ψ*) gradient along the xylem network that allows root-to-leaf water transport, which results in a negative hydrostatic pressure in sap transported through the xylem conduits and implies a risk of xylem embolism formation and propagation (Tyree and Zimmermann 2002).

In past years, knowledge of xylem physiology has improved to the point that it is no longer considered a passive and vulnerable system (e.g. Nardini *et al.* 2011). In particular, several studies have highlighted plants’ ability to modulate their xylem sap composition as a response to external stimuli, and thus affect both hydraulic efficiency (e.g. Nardini *et al.* 2011; Petruzzellis *et al.* 2018; Jupa *et al.* 2019; Oddo *et al.* 2020) and safety (Losso *et al.* 2017; Schenk *et al.* 2017, 2021; Paljakka *et al.* 2020). Changes in the ionic composition of xylem sap, such as increased [K^+^] in response to increased transpiration demand, have been demonstrated to enhance hydraulic conductivity and thus promote water delivery to the leaves (by the so-called ionic effect; e.g. Nardini *et al.* 2011; Oddo *et al.* 2020). Some studies have reported an increase in hydraulic conductivity of more than 25 % upon induced increase in the xylem sap ionic content (i.e. [K+] and [Na+]; López-Portillo *et al.* 2005; Cochard *et al.* 2010; Jansen *et al.* 2011). On the other hand, perfusion with small amounts of Ca^2+^ was shown to suppress any ion-mediated increase in the xylem hydraulic conductance (van Ieperen and van Gelder 2006). Xylem sap pH likely also plays a role in the modulation of the hydraulic efficiency of trees, where pH-induced changes in ions may lead to changes in the overall concentration of carbohydrates in the xylem sap. During drought, xylem sap acidification is thought to enhance the accumulation of sugars in the xylem sap, which might occur from local starch hydrolysis as well as export from the phloem (Secchi and Zwieniecki 2016; Pagliarani *et al.* 2019; Tomasella *et al.* 2019) and may prime the xylem for hydraulic recovery and therefore enhance resistance and resilience against drought-induced stress (O’Brien *et al.* 2014; Tomasella *et al.* 2019).

Xylem sap surface tension (*γ*) has recently attracted interest due to its potential role in the overall hydraulic safety of plants (Losso *et al.* 2017; Schenk *et al.* 2017, 2021; Paljakka *et al.* 2020). Xylem sap *γ* can be altered by the presence of non-surface-active molecules (Andersen *et al.* 1995; Schill and Hartung 1996), such as inorganic salts and sugars that might increase *γ*, and alcohols that might decrease *γ*, as well as by natural surface-active compounds, such as phospholipids, proteins and glycoproteins, known for decreasing *γ* (Iwai *et al.* 2003; Ligat *et al.* 2011; Schenk *et al.* 2017). The presence of insoluble lipid-based surfactants in the xylem sap has recently been hypothesized to increase the hydraulic safety of angiosperms (Schenk *et al.* 2017, 2021), as lipids might coat hydrophobic surfaces and stabilize nanobubbles and thus avoid heterogenous nucleation or air seeding at the pits. However, in alpine conifers, different xylem sap parameters have been shown to vary considerably across seasons (Losso *et al.* 2017, 2018), where xylem sap *γ* reached values much lower than that of pure water (73 mN m^−1^; Tyree and Zimmermann 2002) and might have decreased the resistance to drought-induced xylem embolism of the plants under study (Losso *et al.* 2017).

Knowledge of temporal variation in xylem sap composition and eventual related changes in plant hydraulics is still scarce, with many studies focusing on herbaceous species (e.g. Jackson *et al.* 2003; Wang *et al.* 2012) and only few studies that have investigated variation of multiple xylem sap parameters in tree species across seasons (Bundt *et al.* 1997; Losso *et al.* 2017, 2018; Schenk *et al.* 2021). Information on the ability of plants to modulate their xylem sap composition in response to seasonal changes might help in understanding how trees can cope with the forecast global increase in intense drought events and heatwaves, which are responsible for widespread forest decline and tree dieback (Allen *et al.* 2010, 2015; Arend *et al.* 2021; Nolan *et al.* 2021; Losso *et al.* 2022). This is particularly true for Australia, where 2019 was the third consecutive year of drought and its driest and hottest year on record (e.g. see www.bom.gov.au/climate/data; Nolan *et al.* 2021; Losso *et al.* 2022). Hence, in this study, we focused on six Australian native angiosperm trees and shrubs (*Eucalyptus saligna*, *E. tereticornis*, *E. crebra*, *Hakea dactyloides*, *Petrophile pedunculata* and *Avicennia marina*) to analyse (i) if their xylem sap composition varies across seasons, and (ii) if these changes are interconnected with each other and with hydraulic traits. The selected species are evergreen and thus always active throughout the year. We monitored the percentage loss of hydraulic conductivity (PLC), leaf *Ψ*, xylem sap chemical parameters ([Ca^2+^], [K^+^], [Na^+^], electrical conductivity EC and pH), and xylem sap *γ* over one year (i.e. from December 2019 to December 2020). We expected pronounced seasonal variation in xylem sap composition and relevant effects on measured hydraulic traits from both chemical xylem sap parameters and xylem sap *γ*. In particular, at the peak of the three consecutive years of drought (years 2017–19, see above) effects on plant hydraulics were expected to correspond to changes in xylem sap composition, with particular regard to pH, as xylem sap acidification has often been associated with drought stress in both herbaceous and woody species (Sharp and Davies 2009; Hernandez *et al.* 2016; Losso *et al.* 2018; Tomasella *et al.* 2021). The effects of the drought should be especially obvious when comparing parameters collected at the peak of the drought in December 2019 with those collected during the moderate December 2020.

## Material and Methods

### Plant material

All measurements were performed on branches collected from four evergreen trees and two evergreen shrubs native to Australia and found at three sites situated within the Greater Sydney Area. Three *Eucalyptus* species (*E. saligna*, *E. tereticornis* and *E. crebra*) were growing at the Hawkesbury Forest Experiment (HFE) site situated on an alluvial floodplain near the Hawkesbury River (33°36ʹ40″S, 150°44ʹ26.5″E). The two shrubs (*H. dactyloides* and *P. pedunculata*) were growing near Springwood, in the Blue Mountains (33°42ʹ21.8″S 150°33ʹ26.2″E), and the last tree, a mangrove (*A. marina*), was growing at the edge of the tidal Hawkesbury River in Brooklyn (33°54ʹS, 151°21ʹE).

From December 2019 to December 2020, the study sites were visited monthly at predawn and on each sampling date, four branches (ca. 2 to 3-m-long, from different and randomly selected individuals) per species were harvested. Collections were undertaken before dawn (when potential night-time transpiration is lowest; Zeppel *et al.* 2010, 2011) to ensure full hydration of the stems. Before cutting the branches, four leaves per branch were cut off, placed in small plastic bags, and carried to the lab for water potential (*Ψ*) measurements (20–120 min after sample collection). Then the branch was cut, wrapped in black plastic bags and transported to the laboratory at a constant temperature. From every branch, a side branch (ca. 2-m-long) was cut off under water for percent loss of hydraulic conductivity (PLC) measurements, whereas the rest was used for sap extraction and related analysis (see below). Once in the laboratory (20–120 min after sample collection), all measurements were made within 2–3 h after sampling.

**Figure 1.**
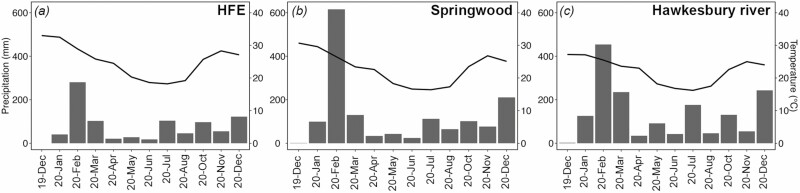
Monthly cumulated precipitation (grey bars) and mean monthly maximum air temperature (black lines) from December 2019 to December 2020 collected from three climate stations situated close by to the three study sites: Hawkesbury Forest Experiment HFE (A), Springwood (B) and the Hawkesbury River (C) (data provided by BOM Bureau of Meteorology).

For each site, mean monthly max temperatures and precipitations were obtained from close-by weather stations (see Fig. 1; Bureau of Meteorology stations 67105, 63077 and 66059; www.bom.gov.au/climate/data).

### Water potential and percent loss of conductivity

Predawn leaf *Ψ* was measured using a Scholander-type pressure chamber (PMS Instrument Company, Albany, OR, USA). We measured four leaves per branch for a total of 16 leaf *Ψ* per species per sampling date. For PLC measurements, branches were placed with their proximal cut end in water and recut to allow relaxation of internal tension (Wheeler *et al.* 2013). For each branch, 4–5 branch segments (2–3 years old and defoliated) were prepared under water, and both proximal and distal ends were trimmed several times with a sharp razorblade to gradually release tension (Wheeler *et al.* 2013) and obtained 8–10 cm long segments. Samples were then connected to a digital liquid flow meter (Liqui-Flow L10, Bronkhorst High-Tech BV, Ruurlo, Gelderland, The Netherlands) and perfused with distilled and degassed water containing 2 mmol KCl and 1 mmol CaCl and filtered to 0.2 μm. The initial hydraulic conductivity (Ki) was measured at a water pressure of 0.002 MPa, and flushing was applied for 15 min at a water pressure of 0.2 MPa to remove embolism. After flushing, the flow rate was measured again (0.002 MPa; final hydraulic conductivity, Kf). Flushing was repeated until measurements showed no further increase in flow rate. All measurements were conducted at room temperature (ca. 21 °C), and PLC (%) was calculated as:


$$\begin{array}{l} PLC=\left(\frac{1\;\;Ki\;}{Kf}\right)\times 100. \end{array}$$ (1)

### Xylem sap composition

For each species under study, xylem sap was extracted and analysed on at least four branches (ca. 3-m-long, collected from different individuals). Once in the laboratory, the proximal end of branches (ca. 5–6 cm) was recut under water with a fresh razor blade and xylem sap extraction was conducted following Schenk *et al.* (2017, 2021). Similarly, the distal end was also cut and the bark was removed (ca. 4 cm length) to expose the xylem cylinder and avoid wound-induced release of solutes into the xylem sap, which was thoroughly cleaned with deionized water using a high-pressure dental flosser (WF-02 Water Flosser, Waterpik Ink, Fort Collins, CO, USA) for 2 min to remove cell debris and cytoplasmic content from the surface. Xylem sap was extracted under vacuum (between −0.080 and −0.095 MPa) by wrapping half of the exposed xylem cylinder next to the bark with Parafilm (Bemis NA, Neenah, WI, USA), and leaving about 2 cm of xylem cylinder freely exposed. The stem was then inserted into a rubber stopper, avoiding any contact between xylem and stopper, and creating a tight seal between Parafilm and stopper. The exposed xylem cylinder was then cleaned again with deionized water as described above and the excess water was removed with a Kimwipe. Xylem sap was directly collected into 4 mL glass vials, which were embedded in ice. Preliminary experiments indicated visible xylem sap extraction started when the stems were cut to 50–60 cm and 30–40 cm above the distal end for trees and shrubs, respectively, as they allowed the applied vacuum to extract the liquid content of the xylem vessels. Hence, after a first cut at about 70 cm (or 50 for shrubs) from the proximal end, a series of subsequent cuts (each 2 cm more distal) from the base upwards were performed, which should lead to a release of xylem sap as soon as the largest vessels were cut open. Once dripping sap was observed, further 1 cm cuts were made every minute to allow for a slow and continuous removal of xylem sap. Depending on stem size, 1–2 mL of sap were extracted from each branch. Extracted xylem sap samples were frozen (−18 °C) until measurements.

For each xylem sap sample, we measured [Na^+^], [Ca^2+^], [K^+^], electrical conductivity (EC) and pH with ion-selective electrodes (Na-11, Ca-11 and K-11 LAQUAtwin Compact Ion Meter), a conductivity metre (EC-22 LAQUAtwin Compact Conductivity meter) and a pH metre (pH-22 Compact pH metre; all from Horiba, Kyoto, Japan). Surface tension (*γ*) was measured for each sample using a modified device based on the pendant drop technique. The main parts of this device consisted of a syringe (Omnifix-F 1 mL syringe with a Sterican blunt cannula; B. Braun Melsungen AG, Germany) to allow a controlled production of drops, a camera (Moticam 3 Plus camera, Motic Deutschland GmbH, Wetzlar, Germany) to take photos of the hanging drops, a light source with a diffuser for maintaining standard light conditions for better image analysis, and temperature sensors to account for temperature-dependent changes in *γ*. For each sample, 5–8 drops were generated and photos were acquired. Image analyses were done using the Pendent Drop plug-in of Fiji (a Java-based distribution of ImageJ, US National Institutes of Health, Bethesda, MD, USA; Daerr and Mogne 2016). This plug-in is based on an internal algorithm that allows to measure the drop profile (i.e. tip radius over capillary length), need for the calculation of the drop *γ*. Xylem sap *γ* was then averaged per each sample.

## Statistics

A principal component analysis (PCA) was used to explore relationships among the recorded xylem sap parameters and hydraulic traits. Differences were tested using one-way ANOVA followed by Tukey’s post hoc comparison (between parameters measured in December 2019 and December 2020). All statistical data were analysed with R 3.6.2 (R Core Team 2017) at a probability level of 5%. All values are given as mean ± SE.

## Results

### Water potential and percent loss of conductivity

**Figure 2.**
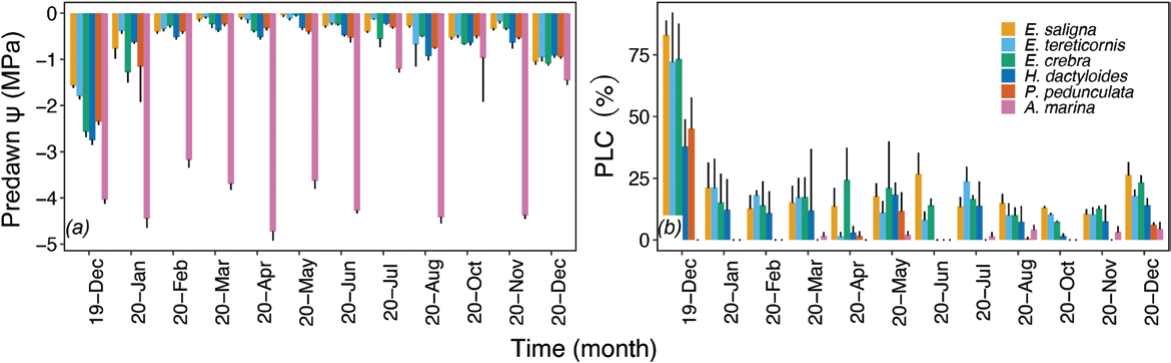
Seasonal variations of predawn leaf water potential (MPa, A) and stem percent loss of conductivity (%, B) of *E. saligna*, *E. tereticornis*., *E. crebra*, *H. dactyloides*, *P. pedunculata* and *A. marina* branches collected every month from December 2019 to December 2020. Mean ± SE, *n* = 3–4.

In all species but *A. marina*, leaf *Ψ* reached lowest values in December 2019 (ca. −2.7 MPa for *E. crebra*, *H. dactyloides* and *P. pedunculata*, whereas ca. −1.8 MPa for *E. saligna* and *E. tereticornis*; see Fig. 2A), which was characterized by high temperatures (>30 °C) and the absence of rainfall (see Fig. 1). In January 2020, leaf *Ψ* increased to values around −1.0 MPa and then became stable with fluctuations between −0.5 and −1.0 MPa for the rest of the year. *Avicennia marina* leaf *Ψ* ranged around −4.0 MPa for most of the seasonal course except for July, October and December 2020 where it reached higher values close to −1.0 MPa (Fig. 2A). For all species but *A. marina*, a similar trend was observed in PLC with the highest values observed in December 2019 (ca. 75 % for the *Eucalyptus* spp., and ca. 45 % for *H. dactyloides* and *P. pedunculata*; see Fig. 2B). In January 2020, PLC decreased and fluctuated around 10–25 % for *Eucalyptus* spp. and *H. dactyloides*. In 2020, *P. pedunculata* PLC values never exceeded 10 %, whereas *A. marina* always had values lower than 10 % in 2019.

A comparison between December 2019 and December 2020 revealed that leaf *Ψ* was significantly lower at the peak of the drought in all species (Table 1). In December 2020, PLC was significantly lower than in December 2019 in the three *Eucalyptus* spp. (Table 1). *Hakea dactyloides* and *P. pedunculata* experienced the same reduction although not significant (Table 1).

**Table 1.** Predawn leaf water potential (MPa), stem percent loss of conductivity (%), xylem sap [K^+^] (mM), [Na^+^] (mM), [Ca^2+^] (mM), electrical conductivity EC (µS cm^−1^), pH and surface tension *γ* (mN m^−1^) of *E. saligna*, *E. tereticornis*, *E. crebra*, *H. dactyloides*, *P. pedunculata* and *A. marina* were measured in December 2019 and December 2020. For each species, asterisks indicate significant differences between months (*P* < 0.05). Means ± SE, *n* = 3–4.

Species	Date	Predawn *Ψ* (MPa)	PLC (%)	[K^+^] (mM)	[Na^+^] (mM)	[Ca^2+^] (mM)	EC (µS cm^−1^)	pH	*γ* (mN m^−1^)
*Eucalyptus saligna*	Dec 2019	−1.57 ± 0.03*	83.10 ± 5.56*	1.44 ± 0.14	0.60 ± 0.05	0.26 ± 0.05	297.00 ± 31.72	6.96 ± 0.02	72.46 ± 0.51
	Dec 2020	−1.05 ± 0.05	26.44 ± 4.88	1.16 ± 0.13	0.45 ± 0.06	0.21 ± 0.01	258.67 ± 28.04	6.37 ± 0.08	71.70 ± 0.41
*Eucalyptus tereticornis*	Dec 2019	−1.80 ± 0.06*	72.34 ± 19.63*	1.51 ± 0.07*	0.53 ± 0.06	0.33 ± 0.09	287.25 ± 13.46	7.16 ± 0.08	69.19 ± 0.59
	Dec 2020	−0.96 ± 0.07	17.89 ± 2.28	1.13 ± 0.07	0.42 ± 0.04	0.31 ± 0.03	270.67 ± 40.28	6.60 ± 0.16	71.16 ± 0.14
*Eucalyptus crebra*	Dec 2019	−2.57 ± 0.12*	73.38 ± 14.01*	2.43 ± 0.4 5*	0.30 ± 0.13	0.59 ± 0.30	413.75 ± 66.61	6.77 ± 0.19	71.29 ± 0.76
	Dec 2020	−1.09 ± 0.03	23.26 ± 2.60	1.42 ± 0.17	0.10 ± 0.01	0.36 ± 0.10	297.33 ± 20.89	6.39 ± 0.08	70.99 ± 0.64
*Hakea dactyloides*	Dec 2019	−2.75 ± 0.10*	37.90 ± 10.68	0.66 ± 0.2	0.51 ± 0.04	0.33 ± 1.03	201.33 ± 23.45	6.95 ± 0.04	69.10 ± 0.64*
	Dec 2020	−0.92 ± 0.03	14.07 ± 2.57	1.21 ± 0.12	0.78 ± 0.07	0.24 ± 0.02	375.33 ± 25.11	6.64 ± 0.15	72.10 ± 1.47
*Petrophile pedunculata*	Dec 2019	−2.35 ± 0.06*	45.23 ± 12.28	0.62 ± 0.02	0.68 ± 0.02*	0.22 ± 0.02	201.00 ± 17.80	6.56 ± 0.07	66.89 ± 1.46*
	Dec 2020	−0.95 ± 0.02	6.22 ± 0.58	0.97 ± 0.11	1.15 ± 0.14	0.26 ± 0.03	255.33 ± 19.97	6.30 ± 0.02	72.00 ± 0.52
*Avicennia marina*	Dec 2019	−4.04 ± 0.07*	0.0 ± 0.0	9.35 ± 0.57 *	34.62 ± 1.65*	1.07 ± 0.13	4920.08 ± 406.20*	6.53 ± 0.18	70.35 ± 0.52
	Dec 2020	−1.46 ± 0.08	4.54 ± 2.50	8.18 ± 1.35	45.53 ± 3.42	1.15 ± 0.24	2058.33 ± 42. 30	6.22 ± 0.15	71.52 ± 0.14

### Xylem sap composition

**Figure 3.**
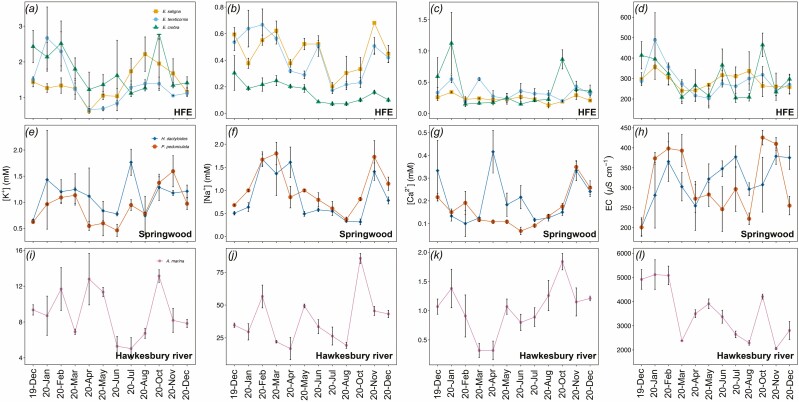
Seasonal variations of xylem sap [K^+^] (mM, A, E and I), [Na^+^] (mM, B, F and J), [Ca^2+^] (mM, C, G and K) and EC (µS cm^−1^, D, H and L) of *E. saligna*, *E. tereticornis*, *E. crebra*, *H. dactyloides*, *P. pedunculata* and *A. marina* branches collected every month from December 2019 to December 2020. Mean ± SE, *n* = 4.

**Figure 4.**
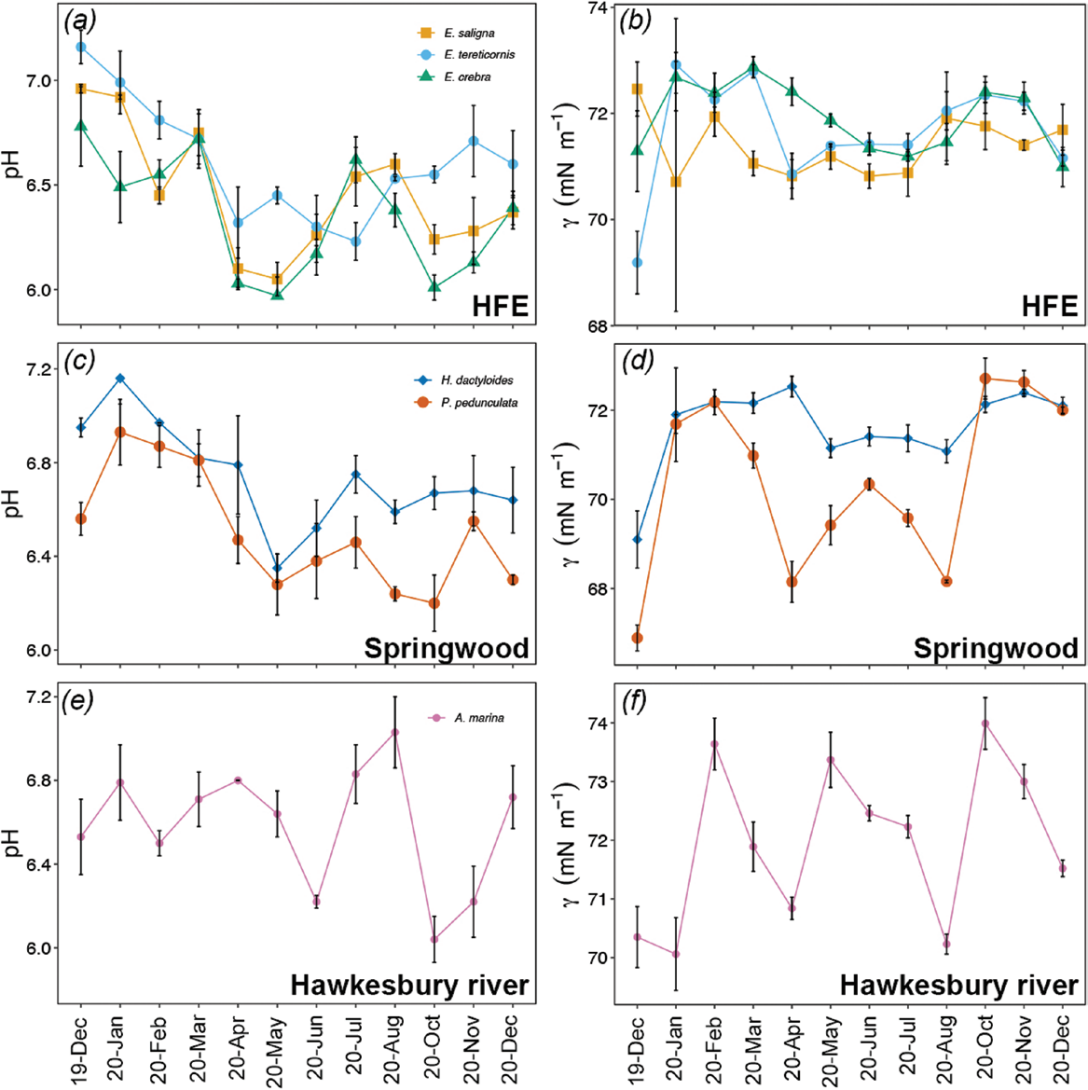
Seasonal variations of xylem sap pH (A, C, and E) and surface tension *γ* (mN m^−1^; B, D and F) of *E. saligna*, *E. tereticornis*., *E. crebra*, *H. dactyloides*, *P. pedunculata* and *A. marina* branches collected every month from December 2019 to December 2020. Mean ± SE, *n* = 4.

In all species, measured xylem sap parameters changed considerably over the annual time course (Figs 3–4). Overall, the ionic composition and EC of all species but *A. marina* were higher in January–March/April and August–November 2020, and lower in May–August 2020 (Fig. 3). In December 2019, ionic concentrations (K^+^, Ca^2+^ and Na^+^) were low in most species except for *E. saligna* and *A. marina*. The latter did not show any clear pattern in xylem sap ionic concentrations except for [Ca^2+^], which decreased at the end of the summer, reaching the lowest values in March–April 2020 (ca. 0.35 mM) to then increasing again towards its maximum values (1.84 ± 0.14 mM, October 2020) and start decreasing again (Fig. 3C, G and K). *Avicennia marina* also had the highest concentrations of both K^+^ and Na^+^ as well as the highest EC, when compared to all the other study species (more than 10-fold higher; Fig. 3).

Xylem sap pH and *γ* also changed considerably over the seasonal course (Fig. 4). In all species, xylem sap pH was between 6.0 and 7.3 with the highest values reported between December 2019 and March 2020 (Fig. 4A, C and E). pH decreased during the winter months and fluctuated for the rest of 2020 with values never exceeding 6.8 (except for *A. marina*, which reached 7.0 ± 0.2 in August 2020; Fig. 4A, C and E). Xylem sap *γ* was overall lowest in *P. pedunculata* with values going as low as 66.9 ± 0.3 mN m^−1^ (December 2019; Fig. 4D). For the other species, xylem sap *γ* ranged between 71 and 73 mN m^−1^, except for December 2019 when *E. tereticornis* and *H. dactyloides* showed their lowest values (69.2 ± 0.6 and 69.0 ± 0.6 mN m^−1^, respectively; Fig. 4B, D and F). The highest xylem sap *γ* values were observed in *A. marina* (74.0 ± 0.4 mN m^−1^; Fig. 4F).

In December 2020, [K^+^] was significantly lower than in December 2019 in *E. saligna*, *E. crebra* and *A. marina* (Table 1). In *P. pedunculata* and *A. marina*, [Na^+^] was significantly higher in December 2020 than in December 2019 (Table 1). *Avicennia marina* also experienced lower EC in December 2020 than December 2019 (Table 1). *Hakea dactyloides* and *P. pedunculata* had significantly higher *γ* in December 2020 over December 2019. [Ca^2+^] and pH did not change between December 2019 and 2020 (Table 1).

**Figure 5.**
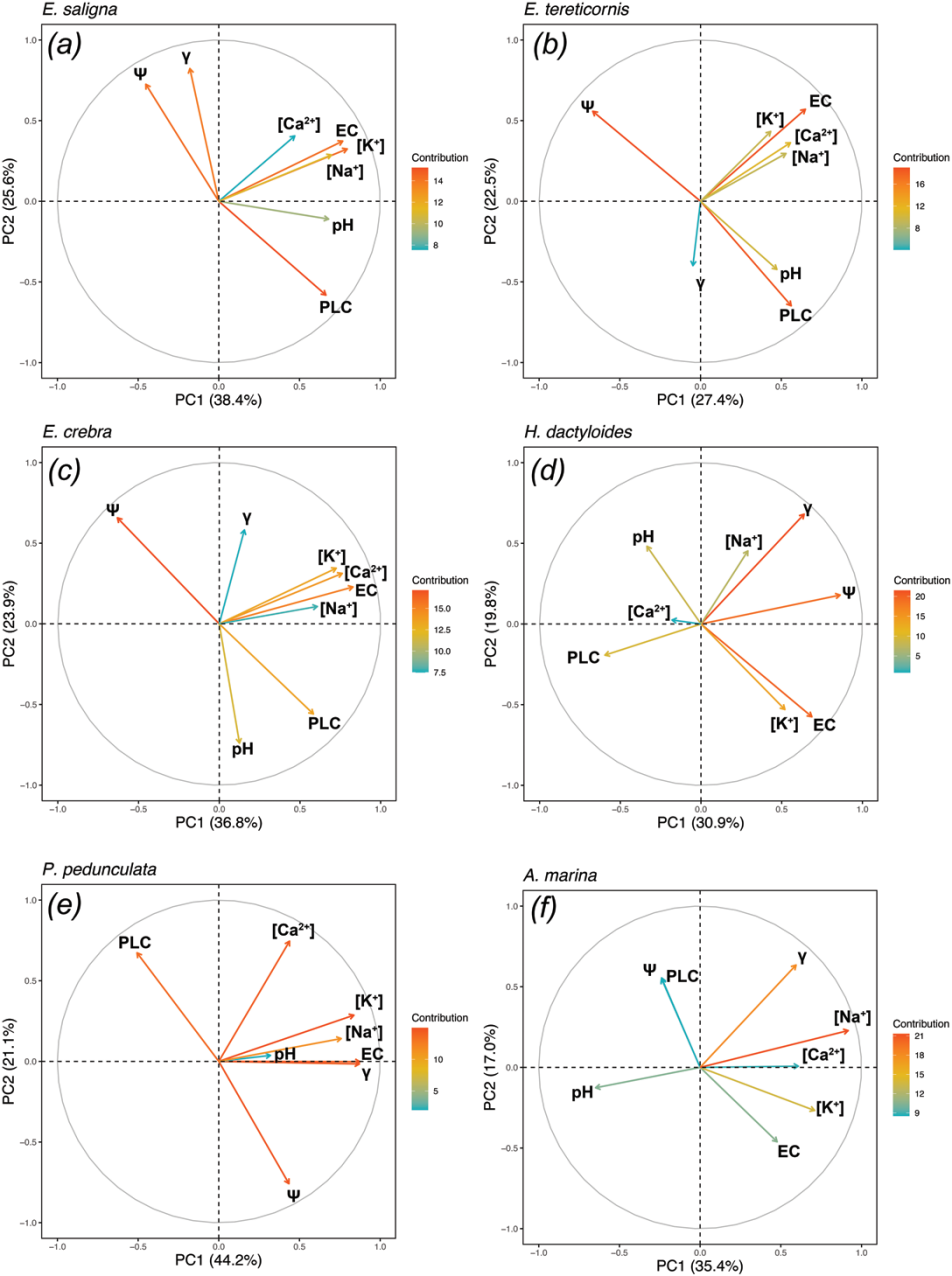
Principal component analysis showing the general associations among xylem sap parameters ([Na^+^], [K^+^], [Ca^2+^], EC, pH and surface tension (*γ*)) of *E. saligna* (A), *E. tereticornis* (B), *E. crebra* (C), *H. dactyloides* (D), *P. pedunculata* (E) and *A. marina* (F) branches collected every month from December 2019 to December 2020. Arrows indicates the contributions of the variables to the principal component axis.

For each species, the relationships among hydraulic traits (PLC and *Ψ*) and xylem sap chemical parameters and *γ* were assessed by performing a PCA. The first two constructed axes (principal components) explained roughly 50–60 % of the variance (Fig. 5). PCAs not only showed several similarities but also some differences between species: for most species (*E. saligna*, *E. tereticornis*, *E. crebra* and *P. pedunculata*), a positive loading was observed for PLC, which corresponded to a negative loading of *Ψ* (i.e. PLC increased while *Ψ* decrease). In *A. marina*, PLC and *Ψ* were both partially negatively loaded. EC and ionic concentrations were positively loaded in all species but *H. dactyloides*, where [Ca^2+^] was negatively loaded (Fig. 5D). *Avicennia marina* showed a strong contribution from [Na^+^] (Fig. 5F). Increasing pH corresponded to a decrease in *Ψ* in *E. tereticornis* and *E. crebra* and in [Na^+^] in *A. marina*. *γ* was strongly positively loaded in *H. dactyloides*, *P. pedunculata* and *A. marina*. Overall, an increase in *γ* corresponded to an increase in *Ψ* (*E. saligna* and *H. dactyloides*), [K^+^] and EC (*P. pedunculata*), and [Na^+^] (*P. pedunculata* and *A. marina*) and to a decrease in in PLC (*E. saligna* and *H. dactyloides*).

## Discussion

Seasonal courses of analysed parameters demonstrated pronounced changes in xylem sap composition and properties, which were likely to be species-specific. In particular, marked differences in xylem sap composition were found between the mangrove *A. marina* and the other species. Contrasting weather conditions during the annual course (i.e. December 2019 at the end of the three consecutive years of drought *versus* the wetter 2020) had an impact on tree hydraulics, while no relevant and consistent changes of seasonal courses in xylem sap composition and properties were observed. However, pH was higher when the hydraulic parameters indicated high levels of drought stress in most plants under study.

In Australia, December 2019 and January 2020 were the end of three consecutive years of extreme drought, with 2019 being the driest and hottest year on record (1.52 °C above the average; www.bom.gov.au/climate/data). This year was then followed by a period of high precipitation which persisted throughout most of 2020, with rainfall above average for much of eastern Australia (14 % above average). At the peak of the drought, our three study sites did not suffer canopy dieback but still experienced above-average temperatures and below-average precipitation (Fig. 1). Accordingly, all species under study except *A. marina*, showed low predawn leaf *Ψ* (<−1.5 MPa) and high values of PLC (>40 %) in December 2019 (Fig. 2 and Table 1). Both hydraulic parameters started recovering from January 2020 reaching and maintaining predawn leaf *Ψ* higher than −1.0 MPa and PLC lower than 20% from February 2020 on (Fig. 2). For *Eucalyptus* spp., full PLC recovery might not have been possible as xylem embolism can potentially persist for several months after rainfall events. As recently demonstrated in *E. saligna* (Gauthey *et al.* 2022), recovery following severe drought may rely on the growth of new xylem rather than embolism refilling and thus occur very slowly. However, after a full year of wet conditions (Fig. 1), every *Eucalyptus* spp. had significantly reduced its PLC (Fig. 2B and 3B). This contrasted strongly with results observed in *A. marina*, with predawn leaf *Ψ* being consistently at about −4.0 MPa throughout most of the year, and PLC close to 0 %. Low leaf *Ψ* is the result of the permanent state of physiological drought that the high salinity of daily tides induces on mangroves (e.g. Ewers *et al.* 2004). With sea water having a *Ψ*_Π_ of about −2.5 MPa, mangroves are forced to maintain their *Ψ* to values lower than those of sea water to avoid water losses and allow soil water uptake (Scholander *et al.* 1965). Higher leaf *Ψ* reported for July, October and December 2020 (>−2.0 MPa; Fig. 2A) is likely the results of the presence of morning dew on leaves (observed during sampling), as mangroves are capable of absorbing external fresh water via their leaves to restore their *Ψ* (Steppe *et al.* 2018; Fuenzalida *et al.* 2019; Schreel *et al.* 2019; Hayes *et al.* 2020).

All investigated chemical components ([Ca^2+^], [K^+^], [Na^+^], EC and pH) as well as the xylem sap *γ* varied substantially during the annual course (Fig. 3–4). In all species, changes within ions and EC were positively correlated (Fig. 5), with an overall tendency towards lower values during winter months (April–August 2020; Fig. 3). Higher [K^+^] during the vegetation period has already been observed in other studies (Trifilò *et al.* 2008; Wang *et al.* 2015; Losso *et al.* 2018). It might improve the overall plant hydraulic conductance (by the so-called ionic effect; e.g. Nardini *et al.* 2011; Oddo *et al.* 2011; Santiago *et al.* 2013) and water-use efficiency (Egilla *et al.* 2005). Two distinct hypotheses have been postulated that might explain the possible principle of the ‘ionic effect’ at pit level: (i) hydrogel theory (e.g. Nardini *et al.* 2011) and (ii) effects of electroviscosity (Santiago *et al.* 2013). A similar pattern was observed in [Ca^2+^], with overall higher values observed during the warmer months (Fig. 3C, G and K). Ca^2+^ ions are important signalling components involved in the regulation of guard cell turgor and therefore stomatal opening and closure (Allen *et al.* 2001; Ng *et al.* 2001).

*Avicennia marina* exhibited disproportionally high concentrations of measured ions when compared to the other species under study (Fig. 3I, J, K and L). *Avicennia marina* is a mangrove growing in intertidal zones affected by daily tides that regularly expose substrates to high salinity, and can withstand high xylem sap salt concentrations and secrete the excess of salt through specialized glands found at the leaf surface (Scholander 1968). Accordingly, Na^+^ was the dominant ion in *A. marina* xylem sap (see Fig. 3B, F and J) as well as the main driver of xylem sap variations (Fig. 5F).

The two shrubs (*H. dactyloides* and *P. pedunculata*) showed more pronounced variation in [Na^+^] with overall higher concentrations when compared to the three *Eucalyptus* spp. (Fig. 3B and F). We see two possible explanations for the higher concentrations compared to studied *Eucalyptus* species: (i) it is a habit-related strategy, as Na^+^ can be used as osmolyte to adjust leaf *Ψ* as reported for mangroves (Suárez and Medina 2008) or (ii) it is a site-related passive effect as both species were growing in the same area.

Measured xylem sap pH values fell within the range reported for other trees (4.5–7.4; Teskey *et al.* 2008; Losso *et al.* 2018) (Fig. 4A, C andE). *Eucalyptus* spp. xylem sap pH (6.0–7.2) was similar to that previously measured in another eucalypt species (i.e. *E. globulus* pH 6.1–7.0; Hernandez *et al.* 2016). In all species, pH was higher during summer 2019–20, decreased during autumn and winter months (May–August 2020), and rose again in spring 2020. In all three *Eucalyptus* spp., changes in pH reflected changes in *Ψ* and PLC, where higher pH corresponded to lower leaf *Ψ* and higher PLC (Fig. 5A–C). Similar results were found for other evergreen species (including *Eucalyptus tetrodonta*) growing in the wet-dry tropics of Australia (Thomas and Eamus 2002), with high pH values also corresponding to higher xylem sap ABA concentrations. These results not only indicated changes in xylem sap pH under water deficiency to be species-specific (Sharp and Davies 2009) but also to be related to the level of drought (Wilkinson *et al.* 1998; Davies *et al.* 2002; Wilkinson and Davies 2002). Under drought stress, increasing xylem sap pH has been documented to enhance ABA loading to the root system and affect the compartmentation of ABA into mesophyll cells in the leaves, which will act as a signal for limiting water loss via stomatal closure (Wilkinson and Davies 1997, 2002; Bacon *et al.* 1998; Davies *et al.* 2002). We observed the highest xylem sap pH values at the end of the three-year-long drought (Fig. 4A and C; see also Fig. 1) when plants likely closed stomata to reduce transpiration. An increase in xylem sap pH might also modulate the hydraulic efficiency of plants under drought stress, as reported by Losso *et al.* (2018) where perfusing stems with solutions at pH 8 enhanced the overall hydraulic conductivity. However, these results are in contrast with other studies suggesting that xylem sap acidification can be used as a proxy of drought stress (Sharp and Davies 2009; Hernandez *et al.* 2016; Tomasella *et al.* 2021), and low pH is thought to enhance apoplastic acidic invertase activity and sucrose hydrolysis (Secchi and Zwieniecki 2016; Pagliarani *et al.* 2019).

Measured xylem sap *γ* varied considerably across the seasonal course and was often lower than that of pure water (73 mN m^−1^; Tyree and Zimmermann 2002) in all species under study (Fig. 4B, D and F). The lowest value was reached by *P. pedunculata* in December 2019 (66.9 ± 0.3 mN m^−1^), whereas the highest by *A. marina* in October 2020 (74.0 ± 0.4 mN m^−1^) (see Fig. 4D and F). Fluctuations in xylem sap *γ* were likely caused by changes in the physiological concentrations of natural surfactants (Iwai *et al.* 2003; Ligat *et al.* 2011; Schenk *et al.* 2017) and, in some species, increasing xylem sap *γ* might have been associated with an increase in the overall ionic concentrations of xylem sap (Fig. 5). Recently, xylem sap *γ* has been demonstrated to play a role in evergreen conifers’ hydraulic safety, where lower xylem sap *γ* would increase the vulnerability to drought-induced xylem embolism (Losso *et al.* 2017; Paljakka *et al.* 2020). However, in our study, xylem sap *γ* did not show any strong correlation with either PLC or *Ψ* (Fig. 5). *Eucalyptus saligna* was the only species in which decreasing xylem sap *γ* was related to decreasing *Ψ* and increasing PLC (Fig. 5A); in *A. marina*, increasing xylem sap *γ* was related to decreasing *Ψ*_Π_ (Fig. 5F).

This study shows that changes in xylem sap chemical and physical parameters are highly complex and dynamic and are likely to vary depending on several external and internal factors. However, despite the high levels of drought stress experienced by the plants under study, xylem sap composition varied mainly according to seasonal changes. pH was the only parameter that increased when PLC increased and *Ψ* decreased and thus might indicate drought responses as signals in the control of stomatal closure and/or as a modulator of the hydraulic efficiency under drought stress. Our results also emphasize the differences in hydraulic traits and ionic concentrations between the mangrove *A. marina* and the non-halophytic land species under study, with Na^+^ being the dominant ion and main contributing parameter in its overall xylem sap variations. Further in-depth studies are required to elucidate possible functional roles of sap chemical and physical parameters for xylem hydraulics.

## Supplementary Material

plad064_suppl_Supplementary_Data

## Data Availability

Raw data from which all figures were generated are provided as Supporting Information. Additional information is available upon request from the corresponding author.
